# Efficacy of HPV Vaccination Regarding Vulvar and Vaginal Recurrences in Previously Treated Women: The Need for Further Evidence

**DOI:** 10.3390/vaccines11061084

**Published:** 2023-06-09

**Authors:** Angela Bechini, Andrea Moscadelli, Beatrice Velpini, Benedetta Bonito, Paolo Orlando, Pasqua Putignano, Silvano Posi, Lorenzo Stacchini, Paolo Bonanni, Sara Boccalini

**Affiliations:** 1 Department of Health Sciences, University of Florence, 50134 Florence, Italy; angela.bechini@unifi.it (A.B.); benedetta.bonito@unifi.it (B.B.); paolo.bonanni@unifi.it (P.B.); 2 Department of Health Sciences, Specialization Medical School of Hygiene, University of Florence, 50134 Florence, Italy; andrea.moscadelli@unifi.it (A.M.); beatrice.velpini@unifi.it (B.V.); paolo.orlando@unifi.it (P.O.); pasqua.putignano@unifi.it (P.P.); silvano.posi@unifi.it (S.P.); lorenzo.stacchini@unifi.it (L.S.)

**Keywords:** human papillomavirus, immunization, genital cancers, cervical lesions, colposcopy, vulvoscopy, HSIL

## Abstract

Vulvar and vaginal cancers are relatively rare cancers, but their incidence is increasing worldwide. Overall, 78% of vaginal cancers and 25% of vulvar cancers are associated with Human papillomavirus (HPV) infection. Immunization could be an option for the management of these cases. We researched and assessed the evidence on the efficacy of HPV vaccination administered to women previously treated with surgery, radiotherapy, or chemotherapy with respect to the recurrence of vulvovaginal disease. From 2006 to November 2022, only one study evaluated the efficacy of HPV vaccination with respect to preventing vulvovaginal recurrences in treated women and showed that a quadrivalent HPV vaccine administered after the surgical treatment of vulvar high-grade squamous intraepithelial lesion (HSIL) can reduce vulvar recurrence of the disease. Therefore, the efficacy of HPV vaccination with respect to vulvovaginal recurrence is still an unexplored field. Further studies are needed to produce stronger evidence in order to appropriately support interventions to protect women’s health.

## 1. Introduction

Human papillomavirus (HPV) is the most common sexually transmitted virus [1]. It is estimated that about 75–80% of sexually active women contract one or more types of HPV during their lifetime [2]. Among women with normal cervical cytological characteristics, the global prevalence of cervical HPV infection is estimated to be 11–12%, for which there are differences between countries [3]. Most HPV viruses cause transient and asymptomatic infections. However, if the infection persists, it can manifest with a variety of lesions, depending on the type of HPV virus [4]. Over 150 different types of HPV viruses have been identified to date [5]. The high-risk HPV types are the main etiological agents of genital tract cancers, such as cervical, vulvar, vaginal, penile, and anal tumors, and a subset of head and neck cancers [6]. 

Cervical cancer, which is the fourth most frequent female cancer, annually constitutes almost 8% of all cancer deaths among women. In fact, 604,127 new cases and more than 341,831 deaths were reported in 2020 [7]. Concerning cervical cancer, some research has highlighted the association between its incidence and case fatality rate and the average income of the population; specifically, about 84% of all cases and 88% of all deaths were observed in low- and middle-income countries (LMIC). Moreover, 70–75% of all cervical cancers and 40–60% of their precursor lesions are caused by HPV types 16 and 18 [8].

Since cervical cancer is a type of tumor that can be thoroughly prevented and effectively treated through vaccination, screening, and therapies, in May 2018, a global call for action was issued by the World Health Organization’s (WHO) Director-General in order to eliminate this burden. In August 2020, the World Health Assembly adopted the Global Strategy for Cervical Cancer Elimination by 2030, which identifies strategic actions necessary to achieve: 90% HPV vaccine coverage for girls by the age of 15 years, 70% coverage for screening, and 90% of women identified with cervical cancer to be treated This is the first global health strategy for eliminating a cancer that has been identified as a public health problem [9].

Vulvar and vaginal cancers are relatively rare tumors worldwide, with 45,240 and 17,908 new cases estimated in 2020, respectively. The rates of both cancers tend to be higher in high-income countries [7]. A high percentage of vaginal cancers are associated with HPV infection (78%), while for vulvar cancer, the percentage of HPV positivity is lower (25%) [10].

Squamous cell carcinoma (VSCC) represents the majority of vulvar cancers, and it can arise through two distinct pathways: HPV-associated and HPV-independent. The precursor of HPV-associated VSCC is vulvar high-grade intraepithelial neoplasia (VIN), which progresses to invasive carcinoma in fewer than 10% of cases but recurs in a higher percentage [11]. A recent systematic review and meta-analysis estimated an aggregate HPV DNA prevalence of 76% in vulvar intraepithelial neoplasia [12]. Although vulvar and vaginal cancers are rare, their incidence has been rising over the past two decades, and this has been partially attributed to the increased development of HPV infection epidemiology [13]. Unlike those for cervical cancer, no screening programs exist for vulvovaginal cancers. Therefore, vaccination programs against HPV represent a priority strategy. 

Several multivalent prophylactic HPV vaccines, which are based on recombinant DNA technology and prepared from purified L1 protein that self-assembles to form virus-like particles, are currently available. The bivalent vaccine includes antigens for HPV 16 and 18 [14]. The quadrivalent vaccine contains antigens for HPV 6, 11, 16, and 18 [15], and the nonavalent vaccine targets HPV types 6, 11, 16, 18, 31, 33, 45, 52, and 58 [16]. These vaccines are extremely immunogenic and lead to the production of specific protective antibodies against HPV [17]. 

The target population includes 9–14-year-old subjects who have been administered two doses of an HPV vaccine and those ≥ 15 years of age who have received three doses. Other recommendations also cover adults who may be at higher risk [18]. A recent study examined the acceptability of HPV vaccination among women aged 25–45 years; among the main reasons for acceptability were protection against the disease, the safety of the vaccine, and the free availability of vaccines, while one of the reasons for refusing vaccination was a lack of information [19].

Evidence shows that prophylactic HPV vaccination, as in the case of cervical cancer precursors, also prevents HPV-related vulvar and vaginal HPV-associated precancers [20,21].

The vaccination of people with previous HPV infection may have less effectiveness, but it prevents new infections with HPV types to which those infected have not been previously exposed. Indeed, having previously been diagnosed or treated for an HPV-related lesion is not a contraindication to vaccination [22]. This is particularly relevant since patients treated for HPV-related high-grade histologic lesions have a significantly increased risk of developing HPV-related malignancies in the 20 years following treatment compared with the general population [23,24]. In addition, several studies have suggested that immunization with HPV vaccines is also effective in preventing new cervical HPV-related lesions in women treated for high-grade squamous intraepithelial lesions (HSIL/CIN2+) and even in women with persistent HPV infection/lesions after treatment [25,26]. Therefore, some European countries [27] have already introduced HPV vaccination for adult women after surgical treatment for cervical lesions.

Many surgical interventions are available as treatment for vaginal and vulvar cancers, some examples of which include ablation, local excisions, vaginectomy, and vulvectomy. In addition, radiotherapy and chemotherapy are also used, which present a variable rate of recurrence after primary treatment [28]. HPV vaccination could also be an option in the management of vulvovaginal disease given its refractory nature, the limitations of screening and diagnosis, and the potentially destructive outcomes of surgery [29]. Therefore, it is important to research and assess the evidence on the efficacy of HPV vaccination with respect to the recurrence of vulvovaginal disease in women previously treated with surgery, radiotherapy, or chemotherapy. 

## 2. Materials and Methods

A literature search was conducted to evaluate the efficacy of HPV vaccination with respect to preventing vulvovaginal recurrences in treated women. Scopus, Pubmed, Clinical Trials, and Embase databases were scoured for this purpose. We considered only clinical trials and observational studies investigating the efficacy of HPV vaccination administered to women after treatment for vulvovaginal disease. Reviews and meta-analyses were excluded in the analysis. The research question of the current study was as follows: “Which is the efficacy/effectiveness of HPV vaccination in preventing recurrence in women treated for HPV-related diseases of vulva and vagina?”.

The keywords “HPV vaccination”, “recurrence”, “vulvo-vaginal neoplasm”, and “treatment” were used in this research and, accordingly, adapted to the format of the different literature databases. We limited our research to articles published in English from 2006 onward. 

Inclusion criteria for the selection of the articles were as follows: manuscripts concerning administration of HPV vaccination after diagnosis or treatment of vulvovaginal lesions and evaluation of the efficacy of HPV vaccination on vulvovaginal recurrence. Outcomes related to cervical recurrence and studies about the prophylactic administration of HPV vaccination were excluded. 

This review was performed in November 2022. The review was independently performed by three pairs of authors. The same pairs of authors screened the titles and abstracts and then the full texts of the papers for eligibility. Any disagreements on the inclusion/exclusion process were resolved in a consensus meeting. We extracted data on the following variables from the studies included in the final synthesis: first author, year of publication, geographical region, aim of the study, study setting, population, type of HPV vaccine administered, outcome type, and type and duration of follow-up.

## 3. Results

The search yielded 606 records from 2006 to November 2022 (260 from Scopus, 131 from Pubmed, 6 from Clinical Trials, and 209 from Embase). After the elimination of duplicates, the remaining number of records was 345. We reviewed the titles and abstracts of all these records and, subsequently, excluded 238 papers because they were not related to the topic of the research (201 papers) or were reviews and meta-analyses (37 articles). Then, we focused on the 107 remaining records, which were reviewed as full texts, and we excluded 106 of them. Specifically 67 articles that did not report topics related to HPV vaccination, 17 articles that reported issues related to HPV vaccination solely for the prevention of cervical disease, 8 articles that reported topics related to HPV vaccination solely for the prevention of warts or anal cancer, 5 articles that did not analyze the eligible population (e.g., analyzing men who have sex with men and/or HIV patients), 4 articles that were not full texts, 3 articles that did not report clinical data, and 2 papers that were conference abstracts were excluded.

**Figure 1:**
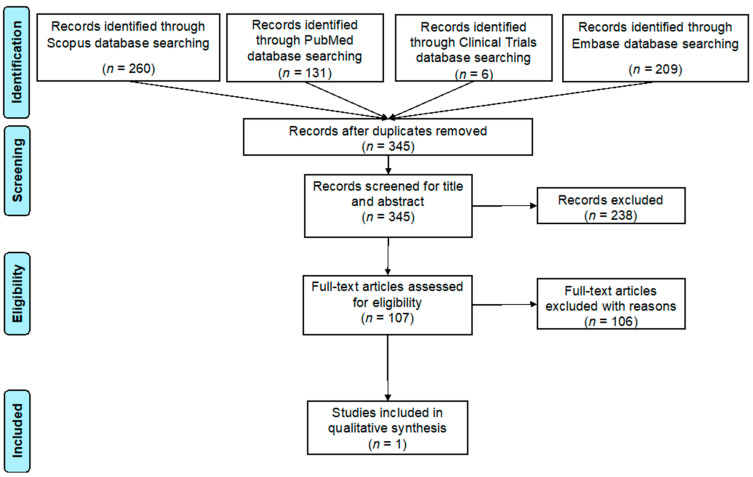
Preferred Reporting Items for Systematic Reviews (PRISMA) flow diagram for the review process.

The selection process of the articles to be included in the final review is provided in the following flowchart (Figure 1). 

Only one study specifically evaluated the efficacy of HPV vaccination with respect to preventing vulvovaginal recurrences in treated women [30]. The manuscript by Ghelardi et al. is a prospective study conducted in Italy and published in 2021. Its results refer to 118 women who underwent surgical treatment for high-grade vulvar squamous intraepithelial lesions (vulvar-HSIL), 42 of whom received the 4-valent HPV vaccine at 0–2–6 months. The study reports a recurrence rate of 19% in vaccinated women compared to that of 32% calculated for unvaccinated women after surgical treatment for vulvar HSIL. While the trend toward a reduction in clinical disease is not significant (*p* = 0.19), clinical effectiveness in preventing a reactivation of the previous HPV infections (relapse) was observed (78.5% of effectiveness). A smaller gap between the groups in terms of recurrence, which was due to the “reactivation” of infection provoked by the same HPV type found during the first surgery, was observed. Therefore, this study supports the impact of vaccination on influencing the natural course of HPV infection after surgery. 

## 4. Discussion and Conclusions

In conclusion, HPV vaccination has a significant role in the prevention of HPV-related diseases. In addition, recent studies have highlighted the effect of vaccination on the management of HPV-related diseases and its impact on recurrent infection or reactivation. However, almost all research conducted until now has mainly focused on cervical disease, while only few studies concern vulvovaginal disease. A recent meta-analysis suggested that the use of adjuvant vaccination is associated with a reduced risk of Cervical Intraepithelial Neoplasia (CIN)1 and CIN2 recurrence after surgical treatment [31]. In fact, several researchers have investigated the role of HPV vaccination in the prevention of cervical recurrences [32,33]. For example, a study conducted in Korea evaluated the efficacy of HPV vaccination among patients undergoing surgical treatment for CIN2/3. According to the results, immunization significantly reduced recurrence in patients with lesions caused by HPV types contained in the vaccine formulations presenting a recurrence rate of 2.5% compared with 8.5% in the unvaccinated group of women [34]. A case–control study conducted in Italy on women treated for CIN2 showed a significantly higher incidence of recurrence in unvaccinated women, namely, 7.9% in the control group compared with 1% in the group of vaccinated women, respectively [24]. Another study conducted in Italy showed that the administration of a vaccine after loop electrosurgical excision (LEEP) among women with cervical dysplasia reduced the risk of recurrence, showing recurrence rates of 16.5% in the control group compared to 7.1% in the group of vaccinated women [35]. All studies concerning the efficacy of HPV vaccination with respect to recurrences are focus on cervical relapse or “de novo” infection. For these reasons, it is now increasingly relevant to evaluate the efficacy of HPV vaccination administered soon after surgery on the recurrence of vulvovaginal disease in treated women. To the best of our knowledge, only the study conducted by Ghelardi et al. [30] investigated the role of HPV vaccination administered after surgical treatment in reducing the incidence of vulvovaginal recurrence. The results of this study show that the HPV quadrivalent vaccine, administered after the surgical treatment of vulvar HSIL, can strongly reduce the vulvar recurrence of the disease. 

Overall, vulvar cancer is more common than vaginal cancer, although it is much less common than cervical cancer. Therefore, one reason for the lack of evidence on this topic could be the low incidence of vulvovaginal cancers [7], which could make it difficult to perform clinical studies on this topic. Even the study by Ghelardi et al. was carried out over a long period on a limited number of treated women [30]. Moreover, the use of small samples could affect the attainment of significant results. Nevertheless, the interest in the potential role of HPV vaccination is important since the incidence of vulvovaginal cancer has been increasing worldwide since the 1970s, which is related to the epidemiology of HPV [13]. Another reason for the interest in this issue is the increased risk of HPV-related vulvovaginal disease among women with a history of cervical disease and the significant risk of vulvovaginal recurrences. Approximately 24% of vulvar cancer cases recur after a primary treatment (surgery with or without radiation) [36]; as for VSCC, recurrent disease occurs in 12–37% of patients [37]. Regarding vulvar or vaginal recurrences, the role of HPV vaccines has been more thoroughly investigated with respect to its use as a prophylactic tool. For example, Joura et al. showed that the incidence of vulvar and vaginal recurrence is lower among women who had received the quadrivalent vaccine before the diagnosis of HPV-related disease [38]. Similar results were obtained in the PATRICIA study on women previously vaccinated with the bivalent vaccine and subsequently undergoing cervical surgery [39]. Moreover, a randomized control trial is currently analyzing the use of a post-surgical HPV vaccine to reduce vulvar and anal HSIL recurrences [40]. 

In addition, vulvovaginal cancer represents a burden for the patients affected and has a high psychological and sexual impact on women and their partners. Considering the causal role of HPV in a large portion of vulvar and vaginal cancers, preventive actions such as the administration of an HPV vaccine have the potential to significantly reduce the burden of HPV disease [41]. Therefore, according to the current knowledge, the efficacy of HPV vaccination with respect to vulvovaginal recurrence is still an unexplored field in contrast to that of cervical recurrence, for which there is more evidence. Consequently, further studies are required in order to obtain more robust evidence with which to appropriately support preventative interventions to protect women’s health.

## Data Availability

No new data were created or analyzed in this study. Data sharing is not applicable to this article.

## References

[1] Ault K.A. (2006). Epidemiology and Natural History of Human Papillomavirus Infections in the Female Genital Tract. Infect. Dis. Obstet. Gynecol..

[2] Chesson H.W., Dunne E.F., Hariri S., Markowitz L.E. (2014). The estimated lifetime probability of acquiring human papillomavirus in the United States. Sex. Transm. Dis..

[3] Forman D., de Martel C., Lacey C.J., Soerjomataram I., Lortet-Tieulent J., Bruni L., Vignat J., Ferlay J., Bray F., Plummer M. (2012). Global burden of human papillomavirus and related diseases. Vaccine.

[4] Boda D., Docea A.O., Calina D., Ilie M.A., Caruntu C., Zurac S., Neagu M., Constantin C., Branisteanu D.E., Voiculescu V. (2018). Human papilloma virus: Apprehending the link with carcinogenesis and unveiling new research avenues (Review). Int. J. Oncol..

[5] Lacey C.J., Lowndes C.M., Shah K.V. (2006). Chapter 4: Burden and management of non-cancerous HPV-related conditions: HPV-6/11 disease. Vaccine.

[6] Garbuglia A.R., Lapa D., Sias C., Capobianchi M.R., Del Porto P. (2020). The Use of Both Therapeutic and Prophylactic Vaccines in the Therapy of Papillomavirus Disease. Front. Immunol..

[7] Sung H., Ferlay J., Siegel R.L., Laversanne M., Soerjomataram I., Jemal A., Bray F. (2021). Global Cancer Statistics 2020: GLOBOCAN Estimates of Incidence and Mortality Worldwide for 36 Cancers in 185 Countries. CA Cancer J. Clin..

[8] Arbyn M., Weiderpass E., Bruni L., de Sanjosé S., Saraiya M., Ferlay J., Bray F. (2020). Estimates of incidence and mortality of cervical cancer in 2018: A worldwide analysis. Lancet Glob. Health.

[9] World Health Organization (2020). Global Strategy to Accelerate the Elimination of Cervical Cancer as a Public Health Problem.

[10] De Martel C., Plummer M., Vignat J., Franceschi S. (2017). Worldwide burden of cancer attributable to HPV by site, country and HPV type. Int. J. Cancer.

[11] Singh N., Gilks C.B. (2020). Vulval squamous cell carcinoma and its precursors. Histopathology.

[12] Li Z., Liu P., Wang Z., Zhang Z. (2023). Prevalence of human papillomavirus DNA and p16INK4a positivity in vulvar cancer and vulvar intraepithelial neoplasia: A systematic review and meta-analysis. Lancet Oncol..

[13] Joura E.A., Lösch A., Haider-Angeler M.G., Breitenecker G., Leodolter S. (2000). Trends in vulvar neoplasia. Increasing incidence of vulvar intraepithelial neoplasia and squamous cell carcinoma of the vulva in young women. J. Reprod. Med..

[14] European Medicines Agency (2022). European Public Assessment Report for Cervarix. www.ema.europa.eu/en/medicines/human/EPAR/cervarix.

[15] European Medicines Agency (2022). European Public Assessment Report for Gardasil. www.ema.europa.eu/en/medicines/human/EPAR/gardasil.

[16] European Medicines Agency (2022). European Public Assessment Report for Gardasil 9. www.ema.europa.eu/en/medicines/human/EPAR/gardasil-9.

[17] Karimi-Zarchi M., Allahqoli L., Nehmati A., Kashi A.M., Taghipour-Zahir S., Alkatout I. (2020). Can the prophylactic quadrivalent HPV vaccine be used as a therapeutic agent in women with CIN? A randomized trial. BMC Public Health.

[18] Kamolratanakul S., Pitisuttithum P. (2021). Human Papillomavirus Vaccine Efficacy and Effectiveness against Cancer. Vaccines.

[19] Mlakar J., Oštrbenk Valenčak A., Kežar J., Beseničar-Pregelj L., Poljak M. (2023). Assessment of Acceptability and Determinants of Uptake and Schedule Completion of Human Papillomavirus (HPV) Vaccine by 25 to 45 Years Old Women in Slovenia. Vaccines.

[20] Joura E.A., Leodolter S., Hernandez-Avila M., Wheeler C.M., Perez G., Koutsky L.A., Garland S.M., Harper D.M., Tang G.W., Ferris D.G. (2007). Efficacy of a quadrivalent prophylactic human papillomavirus (types 6, 11, 16, and 18) L1 virus-like-particle vaccine against high-grade vulval and vaginal lesions: A combined analysis of three randomised clinical trials. Lancet.

[21] Dehlendorff C., Baandrup L., Kjaer S.K. (2021). Real-World Effectiveness of Human Papillomavirus Vaccination against Vulvovaginal High-Grade Precancerous Lesions and Cancers. J. Natl. Cancer Inst..

[22] De Vincenzo R., Caporale N., Bertoldo V., Ricci C., Evangelista M.T., Bizzarri N., Pedone Anchora L., Scambia G., Capelli G. (2021). HPV and Cytology Testing in Women Undergoing 9-Valent HPV Opportunistic Vaccination: A Single-Cohort Follow Up Study. Vaccines.

[23] Pan J., Kavanagh K., Cuschieri K., Pollock K.G., Gilbert D.C., Millan D., Bell S., Graham S.V., Williams A.R.W., Cruickshank M.E. (2019). Increased risk of HPV-associated genital cancers in men and women as a consequence of pre-invasive disease. Int. J. Cancer.

[24] Strander B., Hällgren J., Sparén P. (2014). Effect of ageing on cervical or vaginal cancer in Swedish women previously treated for cervical intraepithelial neoplasia grade 3: Population based cohort study of long term incidence and mortality. BMJ.

[25] Rositch A.F., Soeters H.M., Offutt-Powell T.N., Wheeler B.S., Taylor S.M., Smith J.S. (2014). The incidence of human papillomavirus infection following treatment for cervical neoplasia: A systematic review. Gynecol Oncol..

[26] Ghelardi A., Parazzini F., Martella F., Pieralli A., Bay P., Tonetti A., Svelato A., Bertacca G., Lombardi S., Joura E.A. (2018). SPERANZA project: HPV vaccination after treatment for CIN2. Gynecol. Oncol..

[27] Ministry of Health in Spain (2018). Government Recommendations’ for HPV. www.sanidad.gob.es/profesionales/saludPublica/prevPromocion/vacunaciones/vacunas/ciudadanos/vph.htm.

[28] Stephens S., Chatterjee A., Coles V., Crawford R. (2020). The costs of treating vaginal and vulval cancer in England (2009–2015). BMC Public Health.

[29] Jones R.W., Rowan D.M., Stewart A.W. (2005). Vulvar intraepithelial neoplasia: Aspects of the natural history and outcome in 405 women. Obstet. Gynecol..

[30] Ghelardi A., Marrai R., Bogani G., Sopracordevole F., Bay P., Tonetti A., Lombardi S., Bertacca G., Joura E.A. (2021). Surgical Treatment of Vulvar HSIL: Adjuvant HPV Vaccine Reduces Recurrent Disease. Vaccines.

[31] Di Donato V., Caruso G., Petrillo M., Kontopantelis E., Palaia I., Perniola G., Plotti F., Angioli R., Muzii L., Benedetti Panici P. (2021). Adjuvant HPV Vaccination to Prevent Recurrent Cervical Dysplasia after Surgical Treatment: A Meta-Analysis. Vaccines.

[32] Pieralli A., Bianchi C., Auzzi N., Fallani M.G., Bussani C., Fambrini M., Cariti G., Scarselli G., Petraglia F., Ghelardi A. (2018). Indication of prophylactic vaccines as a tool for secondary prevention in HPV-linked disease. Arch. Gynecol. Obstet..

[33] Del Pino M., Martí C., Torras I., Henere C., Munmany M., Marimon L., Saco A., Torné A., Ordi J. (2020). HPV Vaccination as Adjuvant to Conization in Women with Cervical Intraepithelial Neoplasia: A Study under Real-Life Conditions. Vaccines.

[34] Kang W.D., Choi H.S., Kim S.M. (2013). Is vaccination with quadrivalent HPV vaccine after loop electrosurgical excision procedure effective in preventing recurrence in patients with high-grade cervical intraepithelial neoplasia (CIN2-3)?. Gynecol. Oncol..

[35] Petrillo M., Dessole M., Tinacci E., Saderi L., Muresu N., Capobianco G., Cossu A., Dessole S., Sotgiu G., Piana A. (2020). Efficacy of HPV Vaccination in Women Receiving LEEP for Cervical Dysplasia: A Single Institution’s Experience. Vaccines.

[36] Salom E.M., Penalver M. (2002). Recurrent vulvar cancer. Curr. Treat. Options Oncol..

[37] Nooij L.S., Brand F.A., Gaarenstroom K.N., Creutzberg C.L., de Hullu J.A., van Poelgeest M.I. (2016). Risk factors and treatment for recurrent vulvar squamous cell carcinoma. Crit. Rev. Oncol. Hematol..

[38] Joura E.A., Garland S.M., Paavonen J., Ferris D.G., Perez G., Ault K.A., Huh W.K., Sings H.L., James M.K., Haupt R.M. (2012). Effect of the human papillomavirus (HPV) quadrivalent vaccine in a subgroup of women with cervical and vulvar disease: Retrospective pooled analysis of trial data. bmj.

[39] Garland S.M., Paavonen J., Jaisamrarn U., Naud P., Salmerón J., Chow S.N., Apter D., Castellsagué X., Teixeira J.C., Skinner S.R. (2016). Prior human papillomavirus-16/18 AS04-adjuvanted vaccination prevents recurrent high grade cervical intraepithelial neoplasia after definitive surgical therapy: Post-hoc analysis from a randomized controlled trial. Int. J. Cancer.

[40] Stankiewicz Karita H.C., Hauge K., Magaret A., Mao C., Schouten J., Grieco V., Xi L.F., Galloway D.A., Madeleine M.M., Wald A. (2019). Effect of Human Papillomavirus Vaccine to Interrupt Recurrence of Vulvar and Anal Neoplasia (VIVA): A Trial Protocol. JAMA Netw. Open.

[41] Buchanan T.R., Graybill W.S., Pierce J.Y. (2016). Morbidity and mortality of vulvar and vaginal cancers: Impact of 2-, 4-, and 9-valent HPV vaccines. Hum. Vaccin. Immunother..

